# Shaping Plant Growth Beneath the Soil: A Theoretical Exploration of Fungal Endophyte's Role as Plant Growth‐Promoting Agents

**DOI:** 10.1002/mbo3.70026

**Published:** 2025-07-02

**Authors:** Riyaz Ahmad Rather

**Affiliations:** ^1^ Department of Biotechnology College of Natural and Computational Science Wachemo University Hosaina Ethiopia

**Keywords:** abiotic stress, fungal endophytes, nitrogen, phosphate, plant growth

## Abstract

Plant growth relies on both natural and agrochemical inputs, with natural soil nutrients and chemically synthesized fertilizers enhancing its growth. However, continuous fertilizer use can lead to soil alkalinity and environmental contamination, emphasizing the need for sustainable practices. Microbial agents, particularly fungal endophytes, have emerged as promising natural alternatives. They are recognized as integral components of the plant microbiome and aid in nutrient acquisition, hormone production, and stress resistance. Fungal endophytes enhance nutrient uptake by solubilizing phosphorus, fixing nitrogen, and producing siderophores that chelate iron. They also modulate plant hormones, including auxins, gibberellins, and cytokinins, promoting growth and development. Under abiotic stress, these endophytes improve plant tolerance by inducing systemic resistance and enhancing water and nutrient absorption. This review provides a comprehensive theoretical exploration of the role of fungal endophytes in promoting plant growth, examining their diversity, mechanisms of action, and practical applications. The focus is on understanding how these symbiotic organisms can be harnessed to enhance sustainable agricultural practices and contribute to environmental conservation.

## Graphical abstract

A schematic representation depicting various mechanisms of plant growth promotion by fungal endophytes present in different parts of the plant. Fungal endophytes are present in various parts of the plant, including leaves, stems, and most abundantly in roots. In each of these niches, they promote plant growth through several mechanisms, such as nutrient absorption, hormonal stimulation, nitrogen fixation, iron uptake, and phosphate solubilization. Additionally, they aid in biostimulation and biocontrol mechanisms.

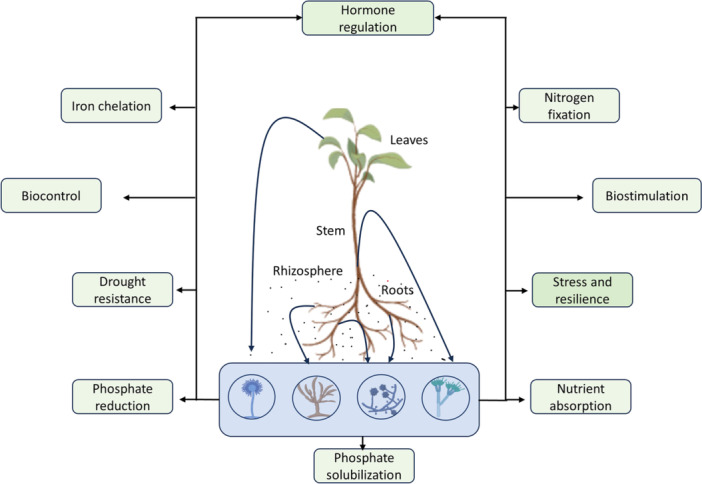

## 1 Introduction

Plant growth is a complex process influenced by both above‐ and below‐ground factors. The availability of sunlight, temperature, precipitation, and environmental conditions are critical above‐ground factors that shape plant development and growth (Jiang et al. 2024). Similarly, nutrients, moisture content, and plant interactions with the rhizosphere significantly impact plant growth (Trivedi et al. 2021). In classical agricultural practices, plant growth is attributed to both natural and agrochemical inputs (Table 1). The nutrient composition naturally available in the soil and chemically synthesized nutrients that are sprayed or mixed with the soil, improve plant growth (Daniel et al. 2022; Yu et al. 2024). Although the latter significantly enhances plant growth and production, it has certain limitations. For instance, continuous use of fertilizers can make the soil alkaline and, in excess, may leach into water bodies, posing an environmental threat (Asif et al. 2025) These chemically synthesized nutrients also carry a significant ecological risk, like, loss of biodiversity (Musah 2024). There is a growing demand for sustainable agricultural practices without further harming the environment. In this context, the exploration of natural and sustainable alternatives has become a focal point of modern agricultural research. Among these alternatives, microbial agents, particularly fungal endophytes, have shown immense promise.

**Table 1:** Comparison of natural and agrochemical inputs in agriculture.

Input type	Benefits	Limitations	Environmental impact
Natural soil nutrients	Improve plant growth naturally.	Limited by soil nutrient composition and availability.	Sustainable and ecofriendly with minimal negative impact.
	Support long‐term soil health.	May not meet the high nutrient demand of intensive farming.	Encourage biodiversity and healthy ecosystems.
	Enhance plant–microbe interactions in the rhizosphere.		
Chemically synthesized nutrients	Significantly enhance plant growth and production.	Continuous use can lead to soil alkalinity.	Potential leaching into water bodies, posing an environmental threat.
	Provide a quick and targeted nutrient supply.	Overuse may harm soil structure and health.	Risk of loss of biodiversity and other ecological risks.
	Can be tailored to specific crop needs.	Can accumulate in the soil, leading to long‐term imbalances.	High carbon footprint associated with production and transportation.
	Provide a quick and targeted nutrient supply.	Overuse may harm soil structure and health.	Risk of loss of biodiversity and other ecological risks.

Explorative research has identified hidden treasures within plants that promote their growth. These hidden treasures, not part of the plant itself but associated with plant tissues, are living symbiotically with plant bodies, and are recognized as endophytes (Ryan et al. 2008; Liao et al. 2025). They are now considered an integral part of the plant microbiome, contributing to plant health and growth. The term endophyte was first introduced in the 19th century, but comprehensive research into their functions and applications has only gained momentum over the past few decades (Mengistu 2020). Today, fungal endophytes are recognized not just as passive inhabitants but as active contributors to plant physiology and growth (Huertas et al. 2024). As endophytes may be of bacterial or fungal origin, this review will explicitly focus on fungal endophytes.

**Figure 1:**
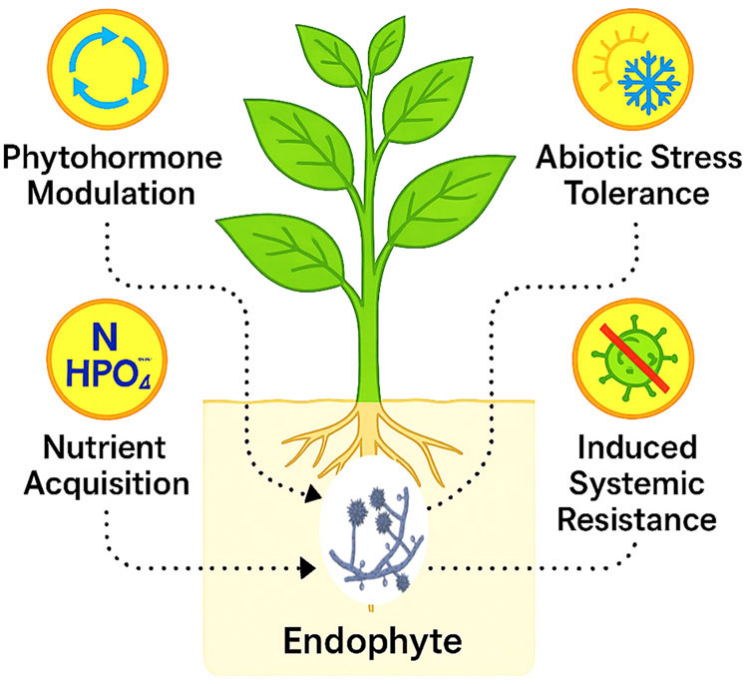
Endophyte and host plant interaction.

Fungal endophytes contribute to plant growth by enhancing nutrient acquisition. They do this through a variety of mechanisms, such as solubilizing phosphorus, fixing atmospheric nitrogen, and producing siderophores that chelate iron (Rana et al. 2020). This makes these essential nutrients more available to the plant. Additionally, they produce plant hormones, such as auxins, gibberellins, and cytokinins, which modulate plant growth and development (Tiwari et al. 2024) (Figure 1). They help plants withstand abiotic stresses such as drought, salinity, and extreme temperatures by inducing systemic resistance and enhancing water and nutrient uptake (Beitsayahi et al. 2025; Kaur et al. 2023). Comparative genomics has revealed that these traits are due to the possession of specific genetic traits that enable them to establish and maintain symbiotic relationships with their host plants. This review aims to provide a comprehensive theoretical exploration of the role of fungal endophytes as plant growth‐promoting agents, examining their diversity, mechanisms of action, molecular underpinnings, and practical applications.

## 2 Fungal Endophyte Diversity and Ecology

Symbiotic relationships between fungal endophytes and plants are key in deciding their ecological niches and diversity. The taxonomic diversity of fungal endophytes spans multiple phyla, including Ascomycota, Basidiomycota, and Zygomycota (Zanne et al. 2020; W. Zhang et al. 2020; Edwards et al. 2025). Irrespective of their varied niche, fungal endophytes display varying degrees of host specificity. Some endophytes are generalists, capable of colonizing multiple plant species, while others are specialists, tightly adapted to a single host species or a closely related group of plants (Zhao et al. 2024).

**Figure 2:**
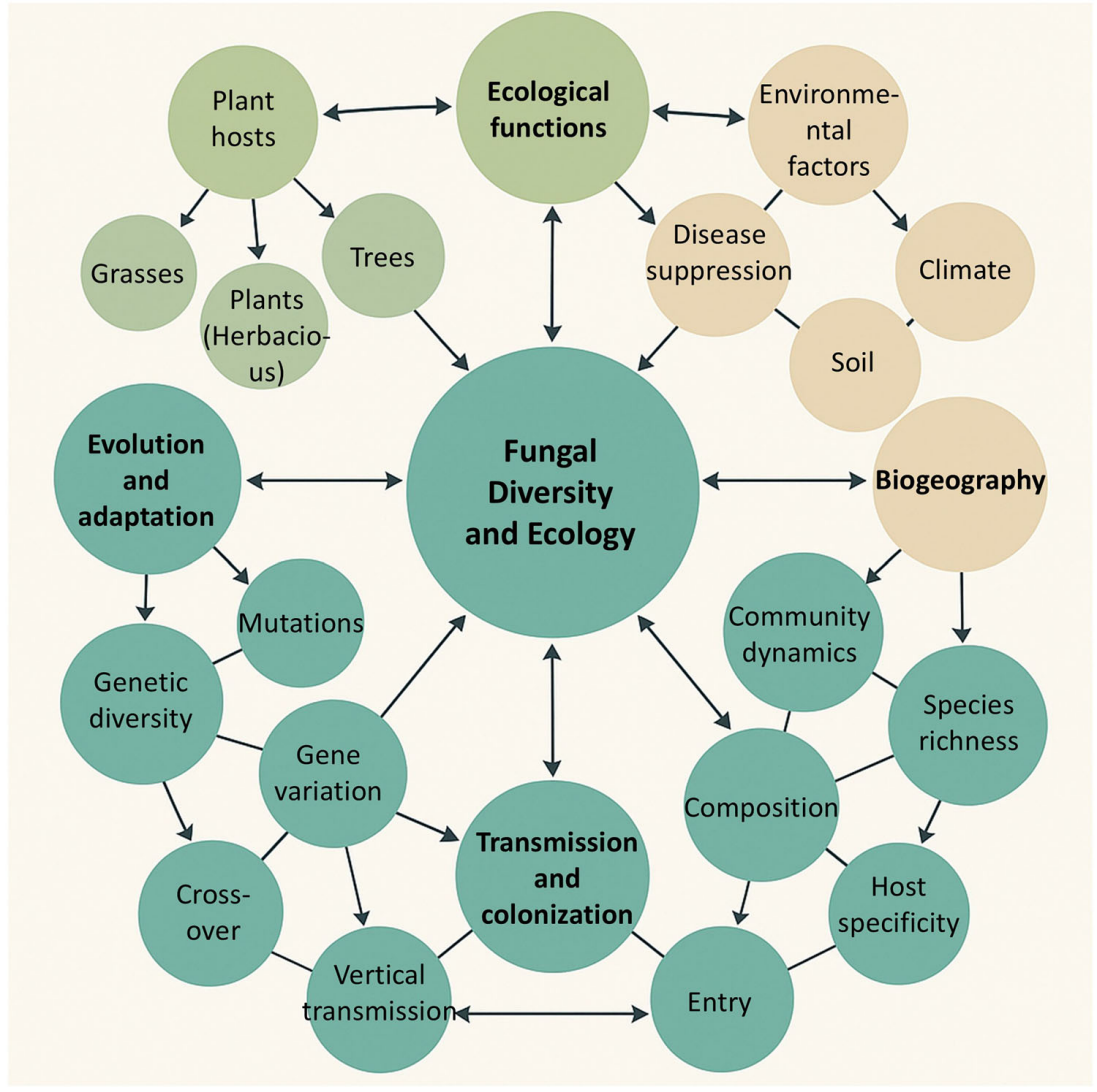
Multidimensional framework of fungal endophyte diversity and ecology. This diagram illustrates the interconnected biological, ecological, and evolutionary components that define fungal endophyte diversity and ecological behavior.

Fungal endophytes occupy distinct niches. These niches include the rhizosphere, phyllosphere, or endosphere (Xu et al. 2022; Gomes et al. 2018; Adeleke and Babalola 2021). In the rhizosphere, fungal endophytes interact with soil microorganisms and plant roots, utilizing the microenvironment of the rhizosphere. They access a rich source of organic compounds exuded by plant roots, which serve as nutrients for the fungi (Deng and Cao 2017). The facilitative effects of endophytic fungi in the plant rhizosphere microenvironment stimulate longer root hairs and increase exudation of secondary metabolites, such as phenolic‐like compounds, into the rhizosphere (Xie et al. 2017). These changes result in more efficient absorption of soil nutrients and enhanced plant–microbe interactions. Whereas, in the phyllosphere, they form symbiotic relationships with the plant's above‐ground tissues, often aiding in defense against pathogens and environmental stressors (Bashir et al. 2022). The endosphere is perhaps the most intimate niche for fungal endophytes. Here, they reside within the plant tissues, often establishing long‐term symbiotic relationships. This niche provides a relatively stable environment, protected from external abiotic stressors (Lu et al. 2021). The endophytic fungi in the endosphere influence plant physiology and metabolism, contributing to growth and stress tolerance. This functional integration is partly mediated by the production of diverse secondary metabolites and signaling molecules that directly affect plant growth, immunity, and nutrient dynamics (Table 2). To further illustrate the multifaceted ecological and evolutionary processes governing fungal endophytes, a conceptual framework is presented in Figure 2.

**Table 2:** Bioactive metabolites produced by fungal endophytes and their role in plant growth promotion.

Fungal metabolite	Produced by fungal genus	Mechanism of action	Benefit to plants
Indole‐3‐acetic acid	*Penicillium*, *Alternaria*, *Aspergillus*	Promotes cell elongation, root initiation, and tissue differentiation	Increased root biomass and architecture complexity
Gibberellic acids	*Fusarium*, *Beauveria*, *Paecilomyces*	Stimulates stem elongation, seed germination, and flowering	Enhanced shoot growth and reproductive output
Siderophores	*Trichoderma*, *Curvularia*, *Phoma*	Chelates iron from soil and facilitates its uptake by plant roots	Improved iron nutrition and chlorophyll biosynthesis
Phytases	*Aspergillus*, *Trichoderma*	Solubilizes organic phosphorus for plant absorption	Greater phosphorus availability and plant biomass
Oxalic acid	*Penicillium, Aspergillus*	Lowers soil pH and mobilizes mineral‐bound nutrients	Improved nutrient solubilization and uptake
Hydrogen peroxide	*Phomopsis*, *Fusarium*	Induces defense signaling and enhances nodulation	Improved nodulation and nutrient assimilation
Nitric oxide	*Phomopsis*, *Epichlo*	Acts as a signal molecule for nitrogen fixation and root development	Enhanced nitrogen fixation and root growth
Brefeldin A	*Colletotrichum*, *Muscodor*	Suppresses pathogen proliferation and supports root health	Protection from fungal pathogens and abiotic stress
Volatile organic compounds	*Muscodor*, *Beauveria*	Enhances systemic resistance and promotes plant vigor	Boosted plant resistance and growth regulation
Cyclopaldic acid	*Phomopsis*, *Glomerella*	Modulates plant immunity and suppresses root pathogens	Improved plant immunity and root system integrity

## 3 Mechanisms of Plant Growth Promotion

### 3.1 Nitrogen Fixation

Nitrogen fixation is a process traditionally associated with symbiotic bacteria such as rhizobia in legumes. However, recent studies have shown that certain fungal endophytes can also contribute to nitrogen fixation. For instance, a study conducted by Xie et al. (2019) demonstrated that the endophytic fungus *Phomopsis liquidambaris* significantly increased root nodulation and N_2_ fixation in *Arachis hypogaea* L. This enhancement was directly correlated with increased peanut yields. In another study, the same endophyte was found to influence nitrogen transformation processes in *Oryza sativa* L., Wuyunjing 7 rice cultivar (Yang et al. 2015). Another study found that increased root nodulation and N_2_ assimilation in *A. hypogaea* was achieved through enhanced hydrogen peroxide and nitric oxide signaling due to the use of the endophytic fungus *P. liquidambaris* (Xie et al. 2017). Similarly, a study by Ongaga et al. (2025) reported that endophytic fungi from nonleguminous plants could fix nitrogen and promote plant growth, suggesting that these fungi could serve as biofertilizers in sustainable agriculture.

**Figure 3:**
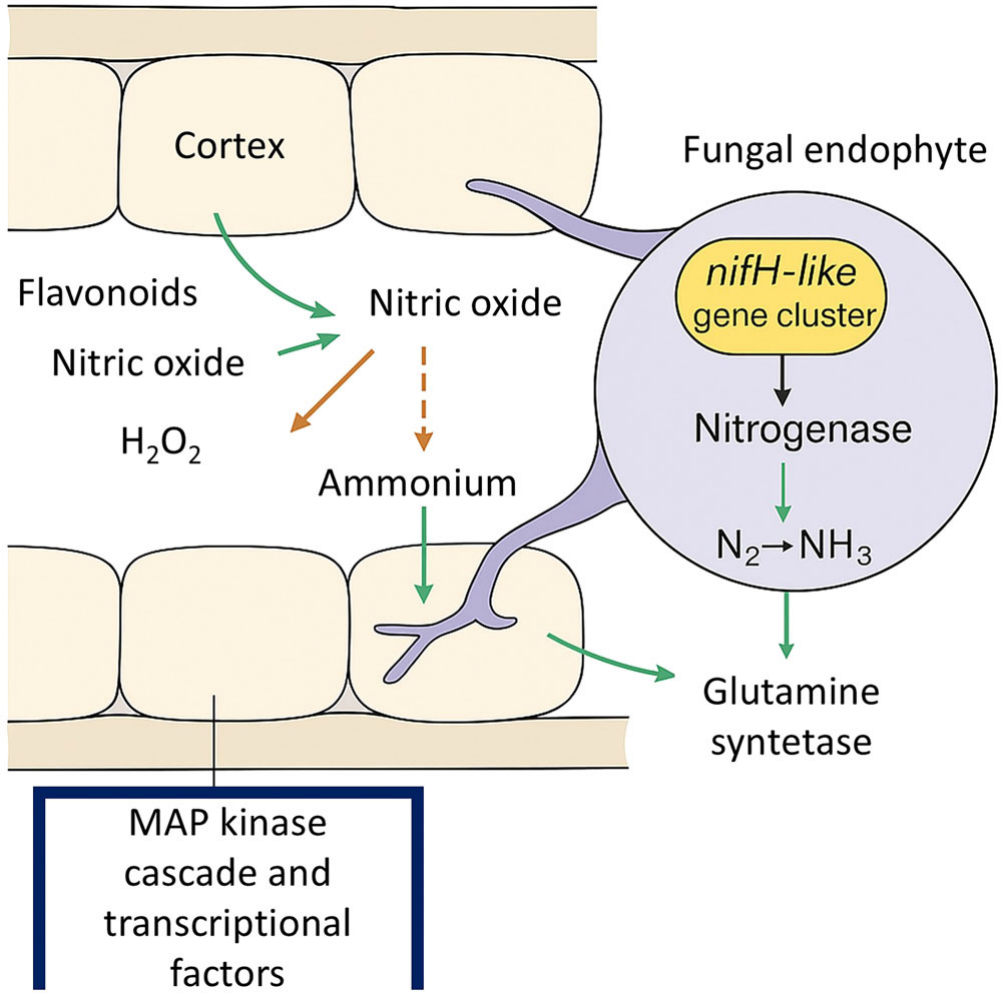
A schematic representation of nitrogen fixation mediated by fungal endophytes in root cortex cells. The fungal endophyte harbors a nifH‐like gene cluster encoding the nitrogenase enzyme complex, which reduces atmospheric nitrogen to ammonia. The generated ammonia is subsequently assimilated into organic forms via glutamine synthetase. Key plant‐derived signals (flavonoids, nitric oxide, or hydrogen peroxide (H₂O₂) modulate the interaction by inducing nitric oxide production, which acts as a signaling molecule facilitating endophyte colonization and nitrogenase activation. Ammonium produced further regulates nitric oxide synthesis, establishing a feedback loop. MAP kinase cascades and transcription factors in host cells coordinate downstream gene expression in response to fungal colonization. MAP, mitogen‐activated protein.

At the molecular level, nitrogen fixation by fungal endophytes is supported by the presence of nifH‐like genes, which encode components of the nitrogenase complex responsible for reducing atmospheric N₂ to ammonia (Mahmud et al. 2020) (Figure 3). These genes, while structurally analogous to those in diazotrophic bacteria, appear to be regulated in a plant‐dependent manner. Transcriptional activation of nitrogenase genes is often linked to nitric oxide signaling and root‐derived flavonoids, which act as molecular cues for the fungal symbionts. In host plants, cross‐communication is facilitated through MAP kinase cascades and transcription factors that upregulate nodulation‐like responses and nitrogen assimilation pathways, even in nonlegumes. This complex regulatory network allows fungal endophytes to integrate into plant root systems, sometimes even enhancing the expression of ammonium transporters and glutamine synthetase in host tissues.

In addition to nitrogen fixation, fungal endophytes enhance nutrient transport within plants. They colonize the root cortex and extend their hyphae into the soil, increasing the root surface area and thereby enhancing the plant's ability to absorb water and nutrients, including nitrogen. This symbiotic relationship is beneficial for the plant, especially under nutrient‐poor conditions. The mycelial network serves as the structural foundation of the mycorrhizal ecosystem, facilitating the dispersal of rhizobia from bulk soil to target plant roots, which subsequently leads to nodulation (W. Zhang et al. 2020). Likewise, endophytic fungi also produce a range of extracellular enzymes and organic acids that solubilize otherwise unavailable nutrients, making it more conducive to nitrogen fixation.

### 3.2 Phosphorus Solubilization

Phosphorus is a critical nutrient for plants, essential for processes such as energy transfer, photosynthesis, and signal transduction. It is present in both organic and inorganic forms. Although phosphorus is abundant in soil, its availability to plants is hindered by factors like imbalanced phosphorus cycling. Some forms of phosphorus despite being present in the soil, are not readily available to plants. Fungal endophytes can convert these unavailable forms into forms that plants can absorb and utilize. They achieve this by producing organic acids and enzymes that mobilize insoluble phosphorus compounds. For instance, citric, oxalic, and gluconic acids produced by fungal endophytes lower the pH in the rhizosphere, which helps dissolve mineral phosphates like calcium phosphate, making phosphorus available to plants (Varga et al. 2020). Likewise, fungal endophytes produce phosphatases, such as phytases, which hydrolyze organic forms of phosphorus in the soil into forms that plants can absorb (Wang et al. 2023). Research by Vassileva et al. (2022) demonstrated that endophytic fungi *Aspergillus*, *Penicillium*, and *Trichoderma* sp., exhibited high phosphate‐solubilizing activity. A group of endophytes exhibiting phenotypic and molecular characteristics similar to *Aspergillus* and *Penicillium* species has been found to induce solubilization of inorganic phosphate in the presence of tricalcium, aluminum, and iron phosphate. These endophytes were isolated from the roots of *Taxus wallichiana* (Adhikari and Pandey 2020). Even dematiaceous endophytic fungi have demonstrated the ability to solubilize Ca_3_(PO_4_)_2_. Arrieta et al. (2015) proposed that the positive association between *Epichloë* endophytes and mycorrhizal fungi enhances phosphate solubilization in the wild grass *Bromus auleticus*. Likewise, research on cucumber has shown that endophytic fungi, specifically *Acer truncatum*, not only enhance phosphorus absorption but also contribute to improved plant biomass and cucumber yield (Zeng et al. 2024).

### 3.3 Iron Chelation (Siderophores Production)

Iron is an essential micronutrient for plants, involved in numerous physiological processes, such as photosynthesis, respiration, and DNA synthesis. However, in many soils, particularly those with high pH, iron availability is limited due to its tendency to form insoluble hydroxides and oxides. Plants have devised various strategies to overcome iron deficiency, like, the secretion of organic acids and the reduction of ferric iron to its more soluble ferrous form. These strategies can be enhanced using fungal endophytes by producing siderophores, which effectively chelate iron and facilitate its uptake by plants (Flores et al. 2025).

Siderophores bind to iron in the soil and form siderophore‐iron complex that is readily transported into plant cells. Once inside the plant, iron is released from the complex through reduction or hydrolysis, thereby becoming available for metabolic processes (Romera et al. 2019). Thus, siderophores produced by fungal endophytes directly contribute to improving iron nutrition in plants. For instance, endophytic fungi *Curvularia protuberataproduce*, *Fusarium culmorum*, and *Phoma* sp., produce hydroxamate‐type siderophores, which are known for their strong iron‐binding properties. Likewise, it has been established that *Trichoderma harzianum* Rifai 1295‐22 produces diffusible chelating metabolites capable of reducing Fe(III) and Cu(II) (Woo et al. 2023).

### 3.4 Hormonal Modulation

Fungal endophytes have a significant impact on plant growth and development through hormonal modulation. The production and regulation of phytohormones such as auxins, gibberellins, and cytokinins by these endophytes play a crucial role in promoting plant growth. Numerous studies have highlighted the mechanisms and effects of this hormonal modulation. For example, a study investigating the effects of endophytic fungus *Serendipita indica* on trifoliate orange (*Poncirus trifoliata*) seedlings revealed significant improvements in plant growth and root architecture (Liu et al. 2023). The inoculation with *S. indica* increased shoot and root biomass, root length, surface area, and volume. Additionally, the concentrations of auxins (indoleacetic acid and indole butyric acid) and cytokinins (trans‐zeatin, dihydrozeatin, and isopentenyl adenine) were significantly elevated in both leaves and roots. This hormonal enhancement was correlated with increased expression of auxin synthesis and transporter protein genes, suggesting that the endophytes improved plant growth and root architecture by modulating endogenous hormone levels. Likewise, another study on common bean (*Phaseolus vulgaris* L.) roots identified endophytic strains, *Alternaria sorghi* and *Penicillium commune*, that produced indole‐3‐acetic acid (IAA) and various gibberellins. These endophytes outperformed exogenously applied hormones in enhancing plant biomass, photosynthetic pigments, carbohydrate and protein contents, and antioxidant enzyme activity (Ismail et al. 2021).

Entomopathogenic fungi that harbor endophytic behavior, including *Beauveria bassiana*, applied to bread wheat (*Triticum aestivum*) seedlings demonstrated how fungal colonization affects plant growth and hormonal gene expression (González‐Guzmán et al. 2022). Initially, these fungi caused downregulation of genes associated with plant immunity and hormone synthesis, leading to a temporary slowdown in growth. However, by 15 days postinoculation, there was an upregulation of auxin‐ and gibberellin‐related genes, suggesting a recovery and subsequent promotion of plant growth (González‐Guzmán et al. 2022). In a similar example, *Aspergillus fumigatus* TS1 and *Fusarium proliferatum* BRL1 isolated from *Oxalis corniculata* roots, were found to secrete IAA and various gibberellins, enhancing the growth of mutant rice (*Waito‐C*) (Bilal et al. 2018). Likewise, *Paecilomyces formosus* isolated from cucumber roots demonstrated significant growth‐promoting effects under salinity stress. This endophyte produced multiple gibberellins and IAA, which enhanced cucumber shoot length and growth characteristics while mitigating the adverse effects of salinity (Khan et al. 2012).

The ability of endophytic fungi to modulate plant hormones and enhance growth has also been reviewed systematically, particularly for woody plants. Endophytes can accelerate plant growth, enhance stress resistance, promote nutrient absorption, and resist pathogens through the production of phytohormones and secondary metabolites (Qin et al. 2024). These interactions highlight the potential of endophytic fungi in improving the adaptability of woody plants to adverse environments.

### 3.5 Stress Tolerance Enhancement

The ability of endophytic fungi to enhance plant growth under stress conditions has garnered significant attention in recent years. The utilization of endophytic fungi presents a sustainable and ecofriendly approach to enhance plant growth and stress tolerance. For example, a study investigating the effects of salt stress on maize revealed that the endophytic fungus *Stemphylium lycopersici* significantly mitigated the adverse impacts of salinity. The association of *S. lycopersici* with maize plants under salt stress conditions resulted in increased antioxidant enzyme activities, enhanced levels of endogenous IAA, and elevated phenolic and flavonoid contents. This symbiotic relationship also led to decreased malondialdehyde (MDA) content and lower concentrations of harmful ions like Na^+^ and Cl^−^, while improving the uptake of beneficial nutrients, such as Ca^2+^, K^+^, and Mg^2+^ (Ali et al. 2022). Endophytic fungi have also shown promise in modulating ion homeostasis in plants under stress. For instance, *Piriformospora indica* colonization in Arabidopsis under salt stress conditions improved plant biomass, chlorophyll content, and lateral root density. The fungus modulated the expression of genes encoding key potassium transporters, thereby maintaining a favorable Na^+^/K^+^ ratio and enhancing the plant's ability to cope with salinity stress (Abdelaziz et al. 2017). The role of endophytic fungi in promoting stress tolerance is not limited to individual fungal strains. Research on the combined inoculation of multiple endophytic strains, such as *Microdochium majus*, *Meyerozyma guilliermondi*, and *Aspergillus aculeatus*, demonstrated synergistic effects on drought‐stressed *Moringa oleifera* (Javed et al. 2022). This consortium significantly improved growth attributes, increased the levels of primary and secondary metabolites, and enhanced antioxidant enzyme activities, leading to better stress tolerance and plant performance. The role of endophytic fungi in promoting nutrient uptake and maintaining ionic homeostasis has been elucidated by Chauhan et al. (2024) on *Aspergillus terreus* in *Vigna radiata*. Under salt stress, plants inoculated with *A. terreus* showed improved root and shoot growth, higher chlorophyll content, and enhanced activities of catalase and superoxide dismutase. Additionally, these plants exhibited reduced proline levels, electrolyte leakage, and MDA content, indicating better stress management and cellular integrity.

Endophytic fungi have been shown to modulate phytohormone levels, thereby enhancing stress tolerance. For instance, *Phoma glomerata* and *Penicillium* sp. were reported to produce biologically active gibberellins and IAA, which significantly promoted growth attributes in rice plants under salinity and drought stress (Waqas et al. 2012). These endophytes also improved nutrient assimilation and reduced sodium toxicity, demonstrating their capability to reprogram host plant growth under stress conditions.

### 3.6 Symbiotic and Antagonistic Interactions

Endophytic fungi are also essential in enhancing plant growth and resilience through symbiotic and antagonistic interactions. These interactions involve mutualistic benefits and the suppression of plant pathogens via mechanisms, such as antibiosis and competition. The plant root is a primary site of interaction between plants and microorganisms, forming the main components of plant microbiomes. The root‐associated endophytic fungi promote plant growth by inhibiting the growth of pathogens and producing secondary metabolites. In some plants, they act as microbial biological control agents that produce antifungal metabolites and compete with pathogens for resources, such as space and nutrients (Vandana et al. 2021). These endophytes exhibit defensive responses like antibiosis, parasitism, lytic enzyme production, and induced systemic resistance in host plants. For instance, *Epichloë*, a genus of filamentous fungal endophytes, forms long‐term symbiotic associations with cool‐season grasses, providing traits, like, pest deterrence and drought tolerance. This genus also exhibits antagonism towards saprophytic and pathogenic microbes, although this trait has been less discussed compared with their mutualistic benefits (Card et al. 2021).

Plant diseases cause approximately 16% losses globally. These losses can be mitigated by endophytic fungi through induced resistance and biological control. It is well established that certain endophytes, such as *Acremonium zeae*, *Muscodor albus*, *Colletotrichum gloeosporioides*, and *Phomopis cassiae*, colonize the deep tissues of various crop plants. These endophytes produce an array of secondary metabolites, including pyrrocidines, brefeldin A, volatile organic compounds, and aciphyllene, among others. These compounds form a biological shield that significantly reduces damage from pathogens and adverse environmental conditions (Bhardwaj et al. 2023).

Competition is another mechanism through which endophytic fungi suppress plant pathogens. By colonizing the same ecological niches within the plant tissues, endophytic fungi outcompete pathogenic organisms for space and resources. This competitive exclusion reduces the opportunity for pathogens to establish themselves and cause disease. For instance, *Beauveria* and *Metarhizium*, endophytic insect‐pathogenic fungi, not only protect plants from insect pests but also compete with soil‐borne pathogens, thereby contributing to overall plant growth.

### 3.7 Biofertilizers and Biostimulants

The formulation of endophyte‐based biofertilizers contains effective fungal strains that can be applied through seed coatings, soil amendments, or foliar sprays. The formulations enhance nutrient uptake and plant development, and also contribute to integrated pest management by bolstering plant resistance to pathogens and pests. Endophytic fungi such as *B. bassiana* have shown promise as biofertilizers and biostimulants. A study conducted at Universitas Andalas investigated the ability of *B. bassiana* to produce IAA and dissolve phosphate, both crucial for plant growth. Moreover, the fungi improved seed germination and plant growth in chili plants (Saragih 2023). Likewise, another study examined the hyperaccumulator plant *Noccaea goesingensis* treated with the endophytic fungus *Phomopsis columnaris* and a seaweed extract‐based biostimulant, Kelpak. The results indicated that while Kelpak alone had no significant effect, inoculation with *P. columnaris* significantly increased the plant's biomass and nickel uptake. The combined treatment yielded even better results, enhancing biomass by 85% and nickel accumulation by 48% compared with untreated plants. This study pinpoints the potential of combining endophytic fungi with other biostimulants to improve phytoextraction efficiency and overall plant growth (Ważny et al. 2021).

Díaz‐Urbano et al. (2023) evaluated the effect of a combination of arbuscular mycorrhiza fungi and *Trichoderma koningii* biostimulant on *Ocimum basilicum* under salinity stress. The biostimulant significantly benefited plant growth, leaf number, and nutrient accumulation under mild salinity stress. It also improved the concentration of beneficial compounds such as polyphenols without affecting the essential oil composition. This indicates that microbial inocula can sustain crop yield and quality even under suboptimal water conditions.

The utilization of biostimulants, including various endophytic fungi, represents a sustainable approach to crop management. By reducing reliance on chemical fertilizers and enhancing plant growth and resilience, these biostimulants contribute to more sustainable and environmentally friendly agricultural practices. The broad host range and diverse mechanisms of action of endophytic fungi make them valuable tools in modern agriculture, especially in the context of climate change and the need for sustainable intensification of crop production.

### 3.8 Case Studies and Application Scenarios

The practical application of fungal endophytes in agriculture has expanded significantly in recent years, with several field‐validated case studies demonstrating. Below we briefly present few field trial cases.

#### 3.8.1 Drought Stress Management

A near recent study evaluated the use of *Trichoderma asperellum* strain T34 as a bioinoculant for maize cultivated under both fully irrigated and drought conditions. The fungus was applied via seed treatment, either alone or in combination with a commercial chemical fungicide (CELEST XL). The T34‐only treatment led to improved physiological performance of maize across both water regimes, including increases in kernel phosphorus and carbon content, kernel number, and kernel dry weight. Under drought stress, T34‐treated plants demonstrated enhanced leaf relative water content, photosynthetic efficiency (PSII maximum efficiency), and water use efficiency, indicating improved drought tolerance. Notably, plants receiving only T34 maintained rhizosphere populations at optimal levels (10⁴–10⁵ CFU g⁻¹ dry soil), which correlated with superior physiological performance compared with the T34 + fungicide combination. These results underscore the potential of *T. asperellum* T34 to serve as a drought‐alleviating agent in maize cultivation, with direct benefits for nutrient accumulation and water stress resilience throughout the crop's developmental cycle (Estévez‐Geffriaud et al. 2020).

#### 3.8.2 Enhanced Phytoextraction Using *P. columnaris* and Seaweed Biostimulants

An experiment examined the application of *P. columnaris* in combination with Kelpak in the hyperaccumulator plant *N. goesingensis*. While Kelpak alone had no significant effect, fungal inoculation resulted in an 85% increase in shoot biomass and a 48% increase in nickel uptake. The combined treatment showed synergistic effects, improving metal solubilization and phytoavailability. This strategy presents an economically viable and environmentally sustainable approach for rehabilitating metal‐contaminated soils, especially in post‐mining regions (Ważny et al. 2021).

#### 3.8.3 Salinity Stress Tolerance in Basil Through Combined AMF and *T. koningii* Application

Another experiment evaluated the impact of a microbial biostimulant containing two strains of arbuscular mycorrhizal fungi and *T. koningii* on *O. basilicum* under low to moderate salinity stress (25 and 50 mM NaCl, respectively). The multispecies inoculum significantly mitigated the adverse effects of salinity by enhancing plant growth, leaf number, and total leaf area, while improving nutrient uptake—particularly of calcium, magnesium, and boron. It also reduced shoot concentrations of nitrate and chloride. Under mild salinity, the inoculum boosted photosynthetic activity through increased availability of iron and manganese, and stimulated the accumulation of phenolic acids, such as caffeic and rosmarinic acids. Under higher salinity, it facilitated sodium sequestration and increased phosphorus availability, while promoting the accumulation of polyphenols like ferulic and chicoric acids and quercetin‐rutinoside. Importantly, the inoculant did not alter the essential oil profile, suggesting that the quality attributes of basil were preserved. This study shows that AMF–*T. koningii* inoculation as an effective strategy for sustaining basil yield and improving stress resilience under suboptimal water quality conditions (Saia et al. 2021).

## 4 Ecological Implications and Biosafety

While fungal endophytes offer substantial benefits as biofertilizers and biostimulants, their large‐scale and long‐term application warrants careful ecological assessment. The introduction of endophytic strains into diverse agroecosystems may inadvertently disrupt the structure and function of native soil microbial communities (Luo et al. 2024). Competitive displacement of indigenous microbiota by introduced endophytes could alter key processes, such as nutrient cycling, organic matter decomposition, and disease suppression. In particular, the dominance of introduced strains may suppress beneficial native symbionts or alter microbial trophic dynamics, leading to unintended consequences for plant–microbe and microbe–microbe interactions (Kong et al. 2025).

Nontarget effects also require serious consideration. Some endophytes may extend their colonization to nontarget plant species, potentially modifying their physiology, growth patterns, or disease susceptibility. Furthermore, endophytic fungi with antagonistic properties might inhibit not only plant pathogens but also beneficial soil organisms, such as mycorrhizal fungi, rhizobia, or phosphate‐solubilizing bacteria (Gao et al. 2025). These indirect ecological interactions are often overlooked but are critical for maintaining functional biodiversity in agroecosystems.

The long‐term ecological stability of endophyte‐based interventions remains underexplored. Repeated application across growing seasons could lead to cumulative shifts in microbial assemblages and biogeochemical fluxes (Chandel et al. 2025). Therefore, rigorous, longitudinal field studies are essential to evaluate the persistence, dispersal, and functional integration of introduced endophytes under various soil and climatic conditions. To mitigate ecological risks, biosafety measures must be integrated into the development and deployment of endophytic formulations. One approach involves engineering biocontainment features such as auxotrophic strains, thereby limiting their survival outside intended settings. Additionally, thorough risk assessment frameworks, incorporating environmental DNA monitoring, metagenomic profiling, and controlled microcosm trials, should be employed before commercial release. By incorporating these ecological considerations and biosafety protocols, the adoption of fungal endophytes can be made more sustainable, reducing the risk of ecological imbalance while maximizing their agronomic potential.

## 5 Challenges and Future Directions

Understanding the specificity of fungal endophytes' interactions with host plants is one of the most challenging aspects of their use. Endophyte–host specificity is regulated by several factors, including the plant's genetic makeup, environmental conditions, soil microbiome composition, and the physiological state of both the plant and the endophyte. Certain endophytes have extensive host ranges and benefit several plant species, whilst others are highly specialized. This specialization can affect the consistency and efficacy of endophyte‐mediated plant growth promotion in a variety of crops and settings. Understanding these interactions at a molecular level is crucial for selecting and using the most effective endophytes in agriculture. The lack of defined criteria for registering and approving microbial bioproducts complicates matters, necessitating regulatory harmonization to encourage wider use. Another significant challenge is ensuring the quality and consistency of products derived from endophytic fungus. Variability in manufacturing techniques, strain selection, and formulation methods might result in variable product performance. Standardized techniques for the isolation, mass manufacturing, and formulation of endophytic fungi are critical for ensuring product efficacy. Quality control techniques, such as strain identity and viability verification, must be thoroughly applied to ensure that endophyte‐based products continuously provide the expected crop benefits.

**Figure 4:**
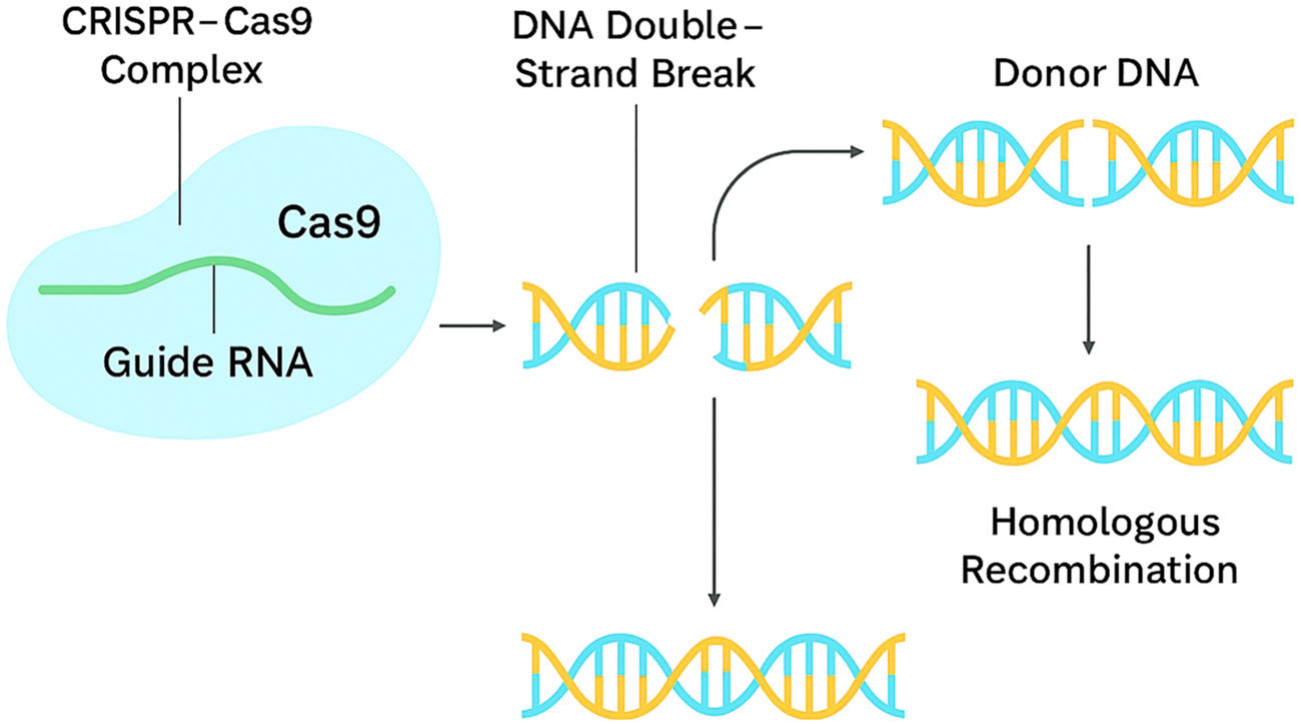
Molecular mechanisms associated with CRISPR–Cas9 genome editing.

Despite tremendous advances, there are still major research gaps in the study of fungal endophytes. The ecological dynamics of endophyte–plant interactions in the field are not well understood, necessitating long‐term research to assess their stability and effectiveness. Additionally, the mechanisms underlying the promotion of plant growth and stress tolerance by endophytic fungi require further elucidation. Identification and characterization of new endophytic strains with potential benefits for a wider variety of crops and environmental circumstances should also be prioritized in research. Advanced biotechnological tools present great opportunities for filling these research gaps and expanding the use of fungal endophytes. Endophytes can be genetically modified using molecular techniques such as Clustered Regularly Interspaced Short Palindromic Repeats‐associated protein 9 (CRISPR‐Cas9), increasing their positive features and making them compatible with a wider range of host plants. While field‐level applications of CRISPR–Cas9 in fungal endophytes are still emerging (Shaizadinova et al. 2025), laboratory studies have demonstrated its potential for trait enhancement, including improved nutrient mobilization (Figure 4). However, the deployment of these engineered endophytes is constrained by regulatory disparities across regions. For example, some countries permit genome‐edited organisms without foreign DNA under relaxed conditions, whereas others apply strict GMO‐equivalent standards. In many developing countries, regulatory guidelines for genome‐edited microbial products remain undefined, which presents a major barrier to field application. Thus, policy harmonization and biosafety validation will be crucial for future deployment. In parallel, metagenomics and other omics technologies can provide complete insights into plant‐associated microbial populations, facilitating the discovery of novel endophytic fungi and understanding their functional roles. These techniques can also assist the establishment of personalized microbial consortia tailored to specific agricultural needs, thus enhancing plant growth and resistance.

## 6 Conclusion

Fungal endophytes present a promising avenue for enhancing plant growth and resilience. These symbiotic microorganisms contribute significantly to plant health by improving nutrient acquisition and enhance stress tolerance. By forming symbiotic relationships with plants, fungal endophytes offer a natural and effective alternative to traditional agrochemicals, which often pose environmental risks and sustainability challenges. Understanding the specificity of endophyte–host interactions is crucial for maximizing their benefits. Continued research and development are essential to fully harness the potential of these microorganisms and integrate them effectively into modern agricultural practices.

## Author Contributions

**Riyaz Ahmad Rather:** conceptualization, writing – review and editing, writing – original draft.

## Ethics Statement

The author has nothing to report.

## Conflicts of Interest

The author declares no conflicts of interest.

## Data Availability

All data discussed are included in the review.
